# A framework for involving coproduction partners in research about young people with type 1 diabetes

**DOI:** 10.1111/hex.13403

**Published:** 2021-12-10

**Authors:** Jane Desborough, Anne Parkinson, Fiona Lewis, Harry Ebbeck, Michelle Banfield, Christine Phillips

**Affiliations:** ^1^ Department of Health Services, Research and Policy, Research School of Population Health, College of Health and Medicine Australian National University Canberra Australian Capital Territory Australia; ^2^ Australian National University Medical School, College of Health and Medicine Australian National University Canberra Australian Capital Territory Australia; ^3^ Centre for Mental Health Research, Research School of Population Health, College of Health and Medicine Australian National University Canberra Australian Capital Territory Australia

**Keywords:** consumer engagement, coproduction, grounded theory, impact, patient and public involvement, patient experience, type 1 diabetes mellitus

## Abstract

**Background:**

Involvement of end‐users in research can enhance its quality, relevance, credibility and legitimacy; however, the processes through which these changes occur are unclear. Our aim was to explore a coproduction research team's experiences of their involvement in research about young people with type 1 diabetes mellitus (T1DM).

**Methods:**

Semi‐structured interviews conducted with two young people with T1DM, two parents, one diabetes educator, one endocrinologist‐scientist and one research‐engineer explored experiences of coproduction research and its impact on both the research and the participants. Drawing on grounded theory, we undertook inductive analysis and storyline mapping to develop a theorized framework of mechanisms supporting the process of coproduction in T1DM research with young people.

**Findings:**

The framework *involving coproduction partners in research about young people with type 1 diabetes* centres on the unique expertize that different team members bring to the research and describes *conditions* that *enable expert contributions* through the enactment of a variety of *expert roles*. The framework also describes *outcomes*—the impact of the expert contributions on both the research and the team members involved.

**Conclusion:**

The findings of this small exploratory study provide a sound foundation to develop further understanding about structures and processes that are integral for the success of coproduction research teams. The framework may provide a guide for researchers planning to incorporate coproduction, on elements that are important for this model of research to succeed. It may also inform coproduction impact assessment research and be used for hypothesis testing and expansion in future studies.

## 1 INTRODUCTION

While there are different perspectives on what involvement in research should look like, the principle of active involvement in research has been adopted broadly by policymakers, researchers and research funding bodies.^1^, ^2^ This includes patient and public involvement (PPI), coproduction, codesign, cocreation and participatory action research, which share philosophical underpinnings and fall under the umbrella of collaborative research.^1^, ^3^ The active involvement of end‐users in the research process, or PPI, has been shown to enhance the quality and relevance of research.^4^, ^5^, ^6^ Studies examining PPI impact have been conducted in the United Kingdom^7^, ^8^, ^9^, ^10^ and Canada.^11^, ^12^, ^13^ In Australia, where the term ‘consumer involvement’ is often used in place of ‘patient and public involvement’,^14^ there is a well‐established body of consumer involvement research.^15^, ^16^, ^17^ In addition to enhancing the scientific and ethical aspects of research, consumer involvement can increase a study's credibility and legitimacy.^18^ Respectful and effective engagement requires the inclusion of numerous and diverse consumer perspectives.^19^

People who draw on their lived experience of a health condition to inform research decision‐making as part of the research team are often referred to as PPI contributors.^12^ Researchers' perspectives on the benefits and challenges of including PPI contributors range from scepticism to enthusiasm and support.^10^, ^20^ Patients and public, and researchers have reported elements that drive PPI, naming shared goals, a supportive environment and provision of feedback as elements for success.^20^

Results of a survey of PPI contributors with type 1 and 2 diabetes found that a critical part of the role was to provide constructive criticism; it was also important to them to receive feedback from researchers.^21^ The involvement of young adults with type 1 diabetes mellitus (T1DM) in research improved understanding of what was needed to enhance health service delivery.^22^ Incorporating teenagers' perspectives and values when designing T1DM interventions is acknowledged as important.^23^, ^24^

Other important end‐users of research are clinicians and policymakers. The inclusion of these and other stakeholders in research, in addition to PPI contributors, is usually referred to as a coproduction model of research rather than PPI alone.^25^, ^26^ Coproduction extends on PPI to ensure that all relevant aspects of a concept are included in research, enabling the construction of complete knowledge,^25^ with the potential to add value to and improve PPI.^27^ The focus is on researchers, PPI contributors and other stakeholders—referred to as ‘co‐production partners’^28^— working together, and sharing decisions and power within a project from beginning to end.^27^, ^28^ Evidence of what does and does not work in the coproduction of research knowledge is scarce.^29^ To our knowledge, no research has examined the experience of including young people with T1DM and their carers, and clinicians in research about young people with T1DM.

Our aim was to explore a coproduction research team's experiences of their involvement in research about young people with T1DM, including their perceptions of the impact of their involvement on themselves and the research itself.

## 2 METHODS

### 2.1 Setting

Our Health In Our Hands (OHIOH) is an Australian National University programme investigating how personalized medicine can address global health challenges, with one key focus on T1DM. The OHIOH Health Experience Diabetes Team (HEDT) is a subgroup whose research focuses on capturing consumers' and healthcare providers' views on personalized medicine and its impacts. Young people aged 12–22 years living with T1DM and healthcare providers are embedded in OHIOH from research inception to implementation (‘living with’ denoting people with T1DM, their carers and families).

The HEDT comprises five people living with T1DM (three young people and two parents), two diabetes educators, one endocrinologist‐scientist and three health services researchers with backgrounds in nursing, sociology and medical anthropology. At the time of writing, for over 2 years, the HEDT has met monthly, sometimes more, to discuss the project, including study design, data collection and analysis. All members contribute to writing papers and grant applications, and are named investigators on these. Some examples of the research and activities conducted by the team are presented in Boxes 1:, 2:, 3:, 4:.

BOX 1: **OHIOH codesign project**

In 2019, the HEDT collaborated with the OHIOH Devices team in codesigning a new breath sensor device aimed to enhance and improve diabetes management. The HEDT met with engineering students and their supervisor on several occasions to provide their perspectives on the characteristics required for a device to be both useable and desirable. Based on their experience, they described preferred physical attributes of a device, such as size and shape, durability and visibility of data. The engineering team used this advice to inform their design—making the device small enough to fit into a pocket, and the data output easily accessible. The engineers returned to subsequent meetings with updated iterations of the device until it was complete.

BOX 2: **OHIOH T1DM cohort research**

A core component of the OHIOH project is a longitudinal cohort study of young people with T1DM. The HEDT worked closely with cohort study researchers in the development of the study protocol for the cohort, including survey design and input into aspects such as the timing, regularity and location of data collection.

BOX 3: **Support for coproduction partners**

The HEDT coproduction partners assisted with data extraction and analysis for a systematic review. To support their capacity to contribute to this, they took part in a 2‐hour session about research methodologies, including systematic reviews. Our project attracted some media attention in 2019 and in anticipation of further contact, the team attended media training arranged by the university.

BOX 4: **Coproduction partners' research and other outputs**

- Coproduction partners were involved in two online conferences in 2020, for which they prepared prerecorded slide presentations.
- Coproduction partners were named coauthors on a published systematic review and a second paper currently under review.
- Coproduction partners gave prerecorded presentations about their work with the HEDT at the 2020 OHIOH symposium.

### 2.2 Design

We adopted a constructivist grounded theory approach for its underlying assumption that reality is influenced by context, within which each individual socially constructs his or her own reality.^30^ Our aim was not to develop theory, but to elucidate the experiences and impact of involving PPI contributors and clinicians in the HEDT, and to explore relationships between these, hence our use of the term ‘grounded theory approach’^31^ also described as grounded theory‐lite.^32^

Grounded theory positions the researcher as one who reconstructs participants' experiences and their meaning through the use of constant comparative analysis, firmly grounding the research in the data (participants' experiences) and findings.^33^ In view of the risk of prior experience and knowledge influencing the outcomes of research, interviews and initial coding were conducted by F. L. who had a background in biology and was a junior medical student with no prior knowledge of the team or of PPI or coproduction. However, she was encouraged to become familiar with these concepts before conducting interviews to stimulate her thinking about the data.^34^ She was trained to conduct interviews by an experienced researcher (M. B.); they conducted the first interview together. Following a reflective exercise to debrief about the experience, F. L. conducted subsequent interviews alone. Interview protocols were designed by two health service researchers (J. D. and M. B.). Other than the student, all researchers had doctoral degrees and qualitative research expertize.

### 2.3 Sample

The sample, approached via email and drawn from the OHIOH HEDT, consisted of two young people living with T1DM and their mothers (PPI contributors), one female diabetes educator from the Canberra Hospital Paediatric Diabetes Clinics (clinician), one male endocrinologist‐scientist from the Canberra Hospital Diabetes Service and a male OHIOH research‐engineer, who was working with the team to design a new device to support diabetes management. While the endocrinologist‐scientist was also a clinician, his primary role in this team was as a researcher. The two teenaged participants (one male and one female) had been diagnosed with T1DM for 2 and 9 years, respectively. Both were managing their diabetes independently with support from their parents if required. One young person was unavailable when interviews were conducted. Two health services researchers from the HEDT (J. D. and A. P.) supervized the student (F. L.) in the conduct of interviews and study analysis, deliberately adopting an arms‐length approach to optimize participants' sense of being able to speak freely during interviews.

### 2.4 Ethical approval

Ethical approval for the study was granted by the Australian National University Human Research Ethics Committee (HREC 2019‐568). Participants provided signed consent to be interviewed and for these interviews to be recorded and transcribed.

### 2.5 Data generation and analysis

Semi‐structured interviews were conducted on the university campus, in two parts. The first explored coproduction partners' (PPI contributors and the diabetes educator) experiences of their involvement in OHIOH and researchers' experiences of involving coproduction partners, and how they felt this impacted both the research and themselves. Crocker et al.^7^ have outlined a typology of impactful roles for PPI contributors, presented in Table 1. We were also interested in exploring whether these roles resonated with the HEDT and if they might identify other roles. In the second part of each interview, participants were provided descriptions of these six roles and were invited to share their perspectives on whether these were reflective of their roles, and if there were additional roles they might identify from their involvement in the OHIOH project.

**Table 1:** Six impactful roles for PPI^7^

Role	Mechanism of impact
The expert in lived experience	PPI contributors are able to consider the acceptability and feasibility of research proposals through the lens of their lived experience
The creative outsider	PPI contributors bring a fresh perspective from outside the research system, and can help to solve problems by thinking ‘outside the box’
The free challenger	PPI contributors are able to challenge researchers without fear of consequences
The bridger	PPI contributors bridge the communication gap between researchers and patients or the public, making research more relevant and accessible
The motivator	PPI contributors increase researchers' motivation/enthusiasm, for example, by emphasizing how the research will benefit people
The passive presence	PPI contributors can change the way that professionals think just by being present at meetings

Abbreviation: PPI, patient and public involvement.

We used constant comparative analysis, amending interview protocols after each interview to iteratively explore concepts as they were identified. This was facilitated through post‐interview discussions between the interviewer (F. L.), who wrote memos after each interview, and supervisors (J. D. and A. P.), whereby concepts were clarified and relevant amendments to subsequent interview protocols were made. Separate protocols were used for PPI contributors and the clinician (Table S1) and researchers (Table S2). Purposeful ordering of interviews facilitated alternative comparison of concepts from different perspectives, enacting Charmaz's assertion that grounded theory can function like a camera—changing one's lens to view scenes from different angles.^33^ For example, the first interview was conducted with a parent, the second with a young person and the third with a researcher, and repeated.

Interviews were of 20–40 min duration and were recorded, professionally transcribed and identifying details were removed. NVivo software^35^ was used to support data storage and analysis. Line‐by‐line coding was conducted by F. L., and used to identify initial concepts, explicit statements and implicit meanings. Initial codes were analysed iteratively for categories and recurrent concepts, resulting in focussed codes.^33^ After identifying and clarifying categories and focussed codes as a team, we used Strauss and Corbin's^34^ coding paradigm based on conditions; actions and interactions; and consequences to support axial coding. On three occasions, the team met and used a whiteboard to identify conditions and mapped out relationships between conditions, the roles and the perceived outcomes. Initially, only roles identified by participants in the first part of interviews were included. Later, these roles and data from the second part of the interviews, where participants described differences from and similarities to those identified by Crocker, were carefully teased out. A theorized, three‐part framework was identified through this process. This inductive approach enabled deep engagement with the data, facilitating interpretation of how specific conditions supported actions and interactions, and participants' perceived outcomes of this process. While analysis of data obtained from the first and main part of the interviews was inductive, analysis of data of participants' perceptions of the six impactful roles was more deductive in that data were examined to elucidate whether participants' experiences in the HEDT described the same roles, synergies with these, or new roles.

We then used the storyline technique^36^ to describe and make further sense of the findings. This iterative process of writing and revisiting the coded interviews enabled refinement of the framework. The student presented the draft framework to study participants at a team meeting and asked whether they believed this was an accurate representation of the process and outcomes of their involvement in the HEDT. Suggested amendments focussed on reducing confusion in the diagram rather than changing content. The team (J. D., A. P. and F. L.) met at a later date and discussed the suggestions, and amended the framework accordingly. This revised framework was again presented to participants, who confirmed that it was an accurate representation of their experiences in the HEDT.

## 3 FINDINGS

Our aim was to explore a coproduction research team's experiences of working on research about T1DM in young people, including the impact on the research and on the team members themselves. Researchers were asked to relate their experiences and the impact of working with coproduction partners, and coproduction partners were asked to relate their experiences of working in the team and their perceived outcomes of this. Our findings provide insight into the breadth of perspectives and dialogues that are brought to this study team through involving a range of stakeholders,^37^ in this case young people with T1DM, their parents, a diabetes educator, an endocrinologist–scientist and a research–engineer. Participants also reflected on the role of other researchers in the team, who were not interviewed for this study.

**Figure 1:**
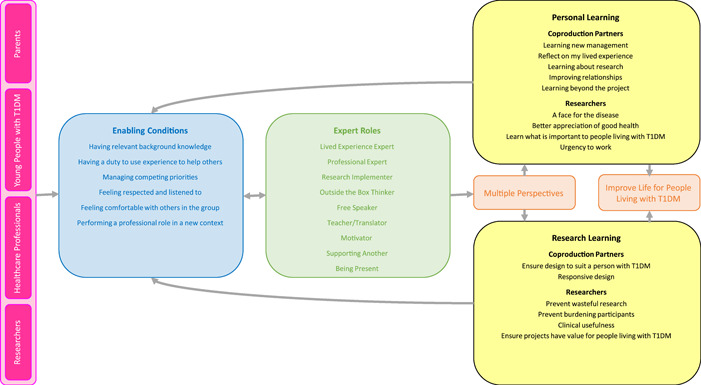
Involving coproduction partners in research about young people with type 1 diabetes

While our aim was not to develop theory, we did theorize a framework, *involving coproduction partners in research about young people with type 1 diabetes*, that participants agreed provides an accurate representation of their experiences in the research about T1DM in young people. The framework centres on the unique expertize that different team members (including coproduction partners and interdisciplinary researchers) bring to the research and describes *conditions* that *enable expert contributions* through the enactment of a variety of *expert roles*. The framework also describes *outcomes*—the impact of expert contributions on both the research and team members involved (Figure 1). Participants described outcomes in terms of personal and research learning for all team members—coproduction partners and researchers. The key outcome arising as a result of their expert contributions to the OHIOH HEDT was perceived as increased potential to improve life for young people with diabetes. The process of *involving coproduction partners in research about young people with type 1 diabetes* was cyclical; both ‘expert roles’ and ‘outcomes’ had the potential to feed back into the conditions that enabled participants to contribute their unique expertize, leading to potential enactment of other ‘expert roles’ and ‘outcomes’. Hence, the process was dynamic—experts (coproduction partners and researchers) could take on new roles and continue learning through their contribution to the team.

### 3.1 Conditions

Participants described six conditions that enabled them to contribute their expertize to the research. Two were related to ‘having relevant background knowledge’ and ‘feeling a sense of duty to use this knowledge and ‘experience to help others’. The third was a practical consideration related to ‘managing competing priorities’. The next two were personal considerations—‘feeling respected and listened to’ and ‘feeling comfortable with others in the group’. The sixth condition referred to the experience of ‘performing a professional role in a new context’.

*Having relevant background knowledge* about diabetes and research affected participants' confidence, and in turn, their readiness to contribute in meetings. Relevant knowledge was not isolated to diabetes; researchers reported feeling more confident when discussing their area of expertize.

*I guess on the topics where I feel comfortable and I feel like, yeah, that influences me, like I'm pretty confident in providing my input*. (P1, Young Person)

*Feeling a duty to use experience to help others* motivated one participant to contribute expertize, to ensure that the resultant device design would not be flawed.

*I don't want other people using it and knowing that there's something I could have changed ‐ that could have made it better*. (P6, Young Person)

This indicated that having expertize of living with T1DM carried a sense of responsibility to people with T1DM more broadly, but such responsibility had to be balanced with *managing competing priorities* for their time. For example, professionals referred to work commitments, whereas the young people referred to sport and homework impacting their capacity to contribute to the project.

*Sport and school occupies most of my life. So I don't really have too much time*. (P6, Young Person)

Young people *felt respected and listened to* in meetings, enabling them to feel free to express their ideas. They knew that their ideas would be considered seriously by the researchers, and this was evidenced by the incorporation of their expert feedback into study protocols and the design of a new device aimed at assisting young people in managing their diabetes.

*From the get‐go it's felt like a safe space and something that we're, a place where I can contribute*. (P1, Young Person)

Who was present at a meeting was a condition that supported participants' contribution, influencing *how comfortable they were with others in the group*, also linked to a sense of respect for their expertize. One health professional expressed an occasional reluctance to speak up due to concern about upsetting perceived hierarchies amongst the various health professionals in the team (e.g., medical vs. allied health professionals). The team's gender mix was mostly female, with one male adult endocrinologist–scientist and one young male. His parent felt that the gender imbalance may have influenced his readiness to participate in some situations, and that this may not have been the case if there had been another young male in the group.

*Performing a professional role in a new context* could be challenging. Being a diabetes educator in a research team rather than working in their career role led to some reticence in contributing, concerned that other members would accept their expert opinion without question. Adaptation to this new role over time enabled this participant to become more comfortable in contributing expertize to the research.

*My role is more just professional advisor as an educator, what my professional experience has been, and what I think is reasonable. (P5, Diabetes educator)*

### 3.2 Expert roles

Participants identified seven expert roles in the first part of the interviews: ‘lived experience expert’, ‘professional expert’, ‘research implementer’, ‘teacher/translator’, ‘being present’, ‘free speaker’ and ‘supporting another’. When discussing the six impactful roles,^7^ they confirmed the ‘motivator’ and the ‘creative outsider’—renaming it as the ‘outside‐the‐box thinker’. One individual affirmed that the six impactful roles directly corresponded to the roles that they already saw present in the team. These roles and participants' reflections are presented in Table 2. Participants believed that one expert role could be enacted by multiple individuals or one individual could enact multiple expert roles.

**Table 2:** Nine PPI contributor roles

Role identified by participants (Crocker et al.'s synonymous role)	Participant reflection/s
Lived experience expert (The expert in lived experience)	*I have the perspective of what it's like living with it… I've got the experience, I'm the one that lives with it… (P6, Young Person)*.
The professional expert	*Their lived experience is their own personal experience, whereas my experience covers many people. (P5, Diabetes educator)*
The research implementer	*When I'm creating something, I've got that right in the back of my head,… they said this, they said that. So, let's do it this particular way that they want. (P4, Researcher)*
Outside the box thinker (The creative outsider)	*[T]hey all have probably a similar thought process, whereas we have the lived experience, but because of that we have the outside the box thinking as well. (P6, Young Person)*
The free speaker (The free challenger)	*They get everyone's opinion; no answer is a wrong answer. You can say something, and someone will be like oh, they'd elaborate with what you'd said. (P6, Young Person)* *[T]hey take on board what you say. They'll ask [the young people] … because they've been made to feel so welcome, [they] will chime in with, you know, whatever they've got to say. (P7, Parent)*
The teacher/translator (The bridger)	*I am the access point to what they're talking about so that… they don't have any exposure to the diabetic lifestyle and things like that and what a diabetic would like in terms of their devices and how we live. (P1, Young Person)* *[The clinician] was probably the bridger between [researchers] and [PLwD] because he knows the science as well as the people with diabetes. (P4, Researcher)*
The motivator	*[OHIOH HEDT leaders] They're more of the motivators in the group, they take the lead. They keep it all good and that. I guess we just follow their lead. (P6, Young Person)*.
Supporting another	*[E]ncouraging and facilitating [my child] to be there, I think that's probably part of my role in providing a space for [name] to participate. (P2, Parent)*
Being present (The passive presence)	*I think just having consumers there does help the researchers actually think like a bit more broadly… [T]here might be weeks where you don't say much at all, there might be other weeks where you have a lot to say. (P7, Parent)*

All participants recognized the value of lived experience. Sharing these experiences was important to ensure that researchers understood the reality of life with the disease, which could generate novel ideas due to their different way of thinking compared with the researchers. The ‘professional expert’ was identified by the diabetes educator and confirmed by others. A professional's knowledge comes from his or her experiences with many others, which provides a different lens. The ‘research implementer’ was identified as an individual who acts on information from meetings and implements it into the research.

*[S]imilar to a bridger, they could make actions about it, do things about it. Be more active with it all*. (P6, Young Person)

Being ‘an outside‐the‐box thinker’ was contingent on the individual having a different and new perspective on the disease, and on their background knowledge, particularly the young people, and researchers from other disciplines, such as the research–engineer. Participants referred to feeling confident to voice their opinions or ‘confident in providing my input’ (P1, Young Person). When presented with the role of ‘free challenger’, they believed that this term encapsulated the role of ‘free speaker’ well, and resonated with participants, who knew that they were invited into the team to bring their different but informed perspective.

Like the ‘bridger’,^7^ the ‘teacher/translator’ was described as someone who explains concepts or communicates between different people present. The role of teacher/translator was the most dynamic of the roles—shifting and changing between individuals depending on the situation and people present in the group. It was the most commonly identified role by participants. No participants identified themselves as the ‘motivator’; instead, they identified the OHIOH HEDT leaders as the ‘motivators’.

One parent identified the role of ‘supporting another’ in relation to their support of their child's participation. ‘Being present’ referred to being present and participating, but not necessarily contributing to the discussion. This has similarities to the ‘passive presence’ role described by Crocker et al.;^7^ however, in our study, it was experienced as an active role. The young people would play this role when they did not know the subject matter; instead, they listened, familiarized themselves with the topic and learned from these discussions. This demonstrated the importance of the condition ‘background knowledge’ in determining whether individuals enacted the ‘being present’ role. Both parents said that just being present at meetings has impact through making the researchers mindful of the young people throughout the research process.

### 3.3 Outcomes

Conditions described by participants enabled them to enact a variety of expert roles—providing advice founded on lived experience of disease and professional experiences as clinicians, engineers and researchers. These expert contributions to research resulted in both personal and research learning. The outcomes gained through involvement in the research positively fed back into the enabling conditions, subsequently increasing the expertize of all involved. At the same time, some participants felt that the team should be expanded to gain other perspectives, such as from even younger‐aged people with T1DM.

#### 3.3.1 Personal learning

For the young people, personal learning was wide‐ranging, including empowering one to change and enhance their management strategies. Another found that learning about other management techniques reinforced their own choices and made them reflect on their own disease experience.

*[L]earning about [other diabetes management] has opened my eyes, and I guess, how I want to manage it in the future and, you know, what the possibilities are to how to manage it… And I'd say that that's from hearing from the other experienced members*. (P1, Young Person)

Participants described the benefits of learning about the research process, and for some, being involved made them consider pursuing further research themselves.

*It's probably highlighted the fact that research is exciting, and I've probably let that go a little bit … [I'm] even thinking of doing my Masters…* (P5, Diabetes educator)

Personal learning was multifaceted and expanded beyond involvement in the project, including having a positive impact on family relationships, and professional development (see Box 3).

*It's given me another way of talking to [name] about diabetes …It's given us more tools to talk openly without being about [them] and [their] management*. (P2, Parent)

*[Y[ou're always learning. … [C]ertainly the media [training] thing the other day, I learnt a heap. And you know, that's not just relevant to research. Like you can translate that to other things, like job interviews and stuff*. (P7, Parent)

PPI contributors personalized the disease for the researchers and fostered a deep connection to the ‘diabetic patient’, enabling them to place a ‘face’ on the disease, and appreciation for good health. *[Y]ou realise how lucky you are not having a chronic disease such as that*. (P3, Researcher)

Working with the young people prompted researchers to consider what is important to people who will use the outcomes of their research, and for one participant, created a sense of urgency, reinforcing the value of their work.

*What it has done is given me a bit more of an urgency … [I]t makes you realise that what you're doing now is actually important*. (P4, Researcher)

#### 3.3.2 Research learning

When discussing their perceptions of the impact of coproduction on the research, participants referred to a codesign aspect of the study, where they had worked closely with the engineering team (see Box 1). An important outcome for PPI contributors was responsive design, where they were able to see their advice and suggestions incorporated into device prototypes. This demonstrated the value of their input and fed into their sense of being listened to, contributing towards their continued readiness to contribute.

*They actually came back, I think it was two months later,… [S]o they gave it to us, show[ed] us how small it was, and they actually said that they'd tested it at one of the university open days and it worked quite well. So it was kind of good to see that it's more coming together now and being more realistic, I guess. It's like I can just see it coming to life*. (P6, Young Person)

When discussing research learning, researchers referred to expert advice provided by the coproduction partners that informed research design (see Box 2). Researchers' learning was multidimensional. Exposure to coproduction partners' perspectives enabled researchers to consider what was valuable to them, resulting in altered research design.

*I think it's incredibly valuable to be able to bounce ideas off them and get their ideas of what we're planning. Is it worthwhile? How will patients actually perceive what we're trying to do here?* (P3, Researcher)

*[What] they do for us is they actually help us define our projects a bit more. After talking to them I've got a bit more [of an] idea*… (P4, Researcher)

Preventing wasteful research was recognized as a key benefit of engaging with coproduction partners to ensure that the research being conducted would be useful to people living with diabetes.

*You might be on the wrong track and it might not be important to, or it might be, you might be dreaming up something that they would never ever dream that they wanted to use or be part of and then you sort of waste a lot of research effort*. (P3, Researcher)

Researchers also came to understand the importance of ensuring that their research was not burdensome for the OHIOH cohort.

*[I]f someone wants to come along and interview all the patients in the cohort like you're interviewing me, you know, just to take into consideration the time commitment… [A]re the questions appropriate, is the interview going to be short, or too long, or overburdening etc? So, that group can give you that feedback…*(P4, Researcher)

Clinical usefulness was a fundamental consideration for those who worked in clinical settings.

*As a clinician, I have my ideas of you know, what, where it might be useful. For example… [If]it flags that, oh this kid might have Type 1 Diabetes and developing ketosis and make an earlier diagnosis rather than them going home—missed diagnosis*. (P3, Clinician)

All participants agreed that the most important outcome was the enhanced capacity to improve life for people living with T1DM.

## 4 DISCUSSION

The coproduction model of research emphasizes equality and the importance of power‐sharing among stakeholders and researchers to foster inclusive research practices and build relationships.^26^, ^38^ At the core is valuing and respecting knowledge from different sources,^38^ which underpins our framework. Our framework describes conditions that optimize contributors' capacity to be involved, and captures value in terms of impact—both identified as gaps in the current literature.^26^ Our findings examine how conditions and roles interact, and how they influence the research and the individuals involved.

### 4.1 Conditions

We identified six key conditions: ‘having relevant background knowledge’, ‘having a sense of duty to use experience to help others’, ‘feeling respected and listened to’, ‘managing competing priorities’, ‘performing a professional role in a new context’ and ‘feeling comfortable with others in the group’. Participants described relevant background knowledge as an essential antecedent to enacting one's expert role/s. The OHIOH team has fostered this for coproduction partners through the provision of new research knowledge and skills, and media training to enhance their capacity for involvement, aligning with previous research indicating the supportive role of such strategies.^11^, ^39^ ‘Having a sense of duty to use experience to help others’ influenced participants' readiness to contribute, supporting previous evidence that committed and engaged participants are a key element to PPI success.^20^ Understanding people's motivation for being involved is important to optimize their effectiveness and potential outcomes.^40^, ^41^

Participants highlighted the benefits of ‘feeling respected and listened to’ and ‘feeling comfortable with others in the group’ as enablers of contributing. A positive, open and trustworthy atmosphere^11^, ^20^ and consumers being respected for their expertize and opinions^18^ are key to successful and sustainable collaboration. The OHIOH team provides remuneration for meeting attendance, which also contributes to the ‘support’ theme identified in previous studies.^11^, ^39^

We included interdisciplinary researchers and clinicians in our research team to ensure that our research was applicable and useable, although on reflection, we realized that in meetings, we tended to primarily focus on the young people with T1DM and their parents—ensuring that their voices were heard and that they were comfortable. PPI and coproduction involve complex social processes^4^, ^42^, ^43^ that may underpin the challenges identified in relation to ‘performing a professional role in a new context’. We were surprised by this finding and that one participant was occasionally reluctant to contribute due to perceived professional hierarchies. This highlighted for us the greater consideration that is needed when implementing a coproduction model, in particular, the need to manage perceptions of hierarchy and power.^28^, ^37^, ^43^ Acknowledgement of this in a large and diverse group is essential, as perceptions of inequality can reduce individuals' motivation to engage.^3^ We subsequently worked to address these concerns through raised awareness and varied meeting configurations. Practical strategies for group discussions such as demonstrating value and respect for diverse views, creating opportunities for quieter voices to be heard and encouraging equality (e.g., using first names instead of official titles) may help address these issues, but require active and constant attention.

Participants referred to the need to manage conflicting priorities, which, for young people, was related to sport and school activities and for professionals, integrating time for their role with work activities. Managing hierarchies and conflicting priorities are acknowledged costs^3^ as teams expand to include more stakeholders.^39^, ^44^ Overall, conditions identified in our study that facilitate PPI mirror those elucidated in previous research, indicating that conditions that enable PPI contribution also enable coproduction.

### 4.2 Roles

We included nine roles in our framework, seven of which were identified independently by participants (lived experience expert, professional expert, research implementer, teacher/translator, free speaker, supporting another and being present), and two previously identified by Crocker et al.^7^ (creative outsider renamed as ‘outside‐the‐box thinker’ and ‘motivator’). Participants acknowledged and confirmed Crocker's six impactful roles—renaming some (Table 2). Several roles were described as pertinent to a variety of team members; for example, the *research implementer* mostly referred to the research–engineer, the *professional expert* referred to the diabetes educator and the *outside‐the‐box thinker* referred to both coproduction partners and the research–engineer. Similarly, the teacher/translator applied to all team members at different times. The roles described are representative of a more holistic understanding of both the context of T1DM and a potential technological solution, reflective of the substantive value of coproduction.^27^, ^37^, ^41^

Our study is unique in its examination of young people's experiences of PPI and, as a consequence, the inclusion of their parents. Most PPI impact studies involve adult patients, with relatively little focus on carers of people with diabetes.^7^ The role ‘supporting another’ was identified by a parent and is unique to our study due to carer involvement in the OHIOH HEDT. One parent reduced their participation to provide more space for their child to contribute. However, carers' lived experience is acknowledged as separate and valuable,^45^ and this parental action to prioritize the child's contribution might have consequentially muffled the parent's voice. Finding a way to balance the needs of both is something that we are considering with our team.

Participants described the roles as fluid; they may alter with changing conditions. The cyclical nature of coproduction in our framework reflects this; roles change over time as participants gain knowledge through personal and research learning, which feeds back into conditions.

### 4.3 Outcomes

Echoing recent research about PPI,^11^, ^20^ participants described distinct benefits of coproduction in terms of both personal and research learning, including the output of research tailored for, and relevant to those involved, to improve life for people living with diabetes. Colearning and research benefits for researchers resulting from PPI have been described previously,^11^ plus therapeutic and education benefits^46^ and enjoyable aspects, such as working towards a common goal.^20^ The outcomes described in our study provide new evidence of the impact of coproduction, advancing knowledge to fill an identified gap.^37^, ^47^

Seeing evidence of their involvement in the research output, including device codesign, was a valued outcome for coproduction partners, feeding back into the condition of ‘feeling respected and listened to’, improving readiness to contribute. Responsive design contributes to a sense of meaningfulness and research relevance for PPI contributors.^20^ A lack of involvement of young people and their caregivers in the design of diabetes technologies has been highlighted,^24^ and the need to improve the depth and breadth of PPI in health technology assessment more broadly.^48^ Our research is addressing these gaps.

Researchers identified *preventing wasteful research, clinical usefulness* and ensuring that *projects have value for people living with T1DM* as key outcomes in this study. The latter two may be considered to be aspects of preventing wasteful research, the cost of which has been highlighted and substantial efforts have been made to reduce this in recent years.^49^ Our findings elucidate how coproduction can address issues of waste,^49^ in particular by optimizing the quality of the design and conduct of research, enhancing its impact on research, clinical practice and policy.

Our findings also report impact on the people involved in the research, some of whom will be end‐users. The true impact of our research on policy and practice will be tested when we work to implement the outcomes into clinical practice, including the device being codesigned by our team members. We aim to facilitate this through continued involvement of clinicians and the local health service, requiring consideration of our needs in terms of advocacy skills and tools.^37^

### 4.4 Other considerations

The nature of working with young people is that they grow up and seek different paths. Similar to recent research involving young people with T1DM,^44^ it will be important for us to continue our relationships with current members as they grow, and to also engage with and nurture younger members. Participants reported subjective experiences and short‐term outcomes. We acted on their suggestion to broaden the team expertize, now including a younger male with T1DM—a medium‐term outcome. Participants described personal learning of transferable skills such as data analysis, media presentation skills, grant writing and research dissemination, also described in recent research,^44^ which may be described as long‐term impacts. From a research perspective, the long‐term impacts of coproduction in this study about young people with T1DM will also describe the impact on the research agenda and culture for the broader project and within the university, including when, where and how it can be most effective.^26^ These represent valid ways of understanding the impact of coproduction in this study.^50^

### 4.5 Future directions

Our experience affirms that coproduction is hard but rewarding work.^3^, ^28^, ^37^ Involving people living with T1DM in research requires an organisational commitment to fund this activity and create conditions conducive to PPI contribution, which we have worked to embed into our project. Our experience of involving clinicians and interdisciplinary researchers in addition to PPI contributors has been that it requires even greater active and continual outreach and engagement. A dedicated support position is required to support this ongoing engagement in large projects.

A variety of frameworks for supporting PPI in research exist and include similar components to our framework; however, the literature is not clear on how such elements can be combined to understand how these processes work together. We believe that our findings and our framework make a valuable contribution to understanding this and can inform future research embedding either PPI alone or coproduction. At the same time, we acknowledge that there is no ‘one‐size fits all approach’ and challenges and enablers to coproduction will vary with context.^43^ It takes time and commitment to build relationships and capacity, and to create a team that can work together in a safe space where different perspectives and opinions can be aired. There is a need for studies that focus on the effect of coproduction and tests of when, where and how it can be used most effectively. We hope our study will contribute to this new knowledge.^37^

## 5 VALIDITY AND RELIABILITY

Regular, iterative analysis at weekly team meetings ensured the methodological rigour of this study, in particular, the determination of codes and categories as they were identified and how to explore these. This enhanced the transferability of our findings by incorporating contributions from debriefing following interviews into the subsequent interviews and ultimately into the framework.^51^ Incorporation of feedback from the OHIOH HEDT into the final framework established credibility.^51^

## 6 LIMITATIONS

Our study reports the experiences of a small team of researchers focusing on T1DM. Due to the finite size of our sample, we were unable to establish if we had reached data saturation, although the analysis yielded enough insight to inform a theoretical model. Confirmation of our model with another team inclusive of research contributors will increase the transferability of our framework, confirming its applicability to different groups and settings.^51^ The majority of people involved in this study were closely linked to the project, which may have added an element of bias.

## 7 CONCLUSION

The outcomes or impact of coproduction research are shaped by many contextual factors, including the skill and experiences of the people involved, and the subject matter.^42^ Understanding the mechanisms that operate in specific contexts to produce particular outcomes can inform research planning. Our findings contribute new evidence to the understanding of conditions that enable the contribution of unique and broad expertize to research and outcomes arising from this. While this was a small exploratory study, it provides a sound foundation to further develop understanding, and also tools to support coproduction research. The framework may provide a guide for researchers planning to incorporate coproduction, on elements that are important for this model of research to succeed. It may also inform coproduction impact assessment research and be used for hypothesis testing and expansion in future studies.

## CONFLICT OF INTERESTS

The authors declare that there are no conflict of interests.

## AUTHOR CONTRIBUTIONS

Jane Desborough: *Conceptualisation* (ideas; formulation or evolution of overarching research goals and aims [including preparation and submission of ethics protocol]). Jane Desborough, Christine Phillips, Anne Parkinson, Fiona Lewis, Michelle Banfield: *Methodology* (development or design of methodology, including interview protocol; creation of models). Fiona Lewis, Jane Desborough, Anne Parkinson (NVivo coding and confirmation of codes): *Software* (programming, software development; designing computer programmes; implementation of the computer code and supporting algorithms; testing of existing code components). Anne Parkinson, Jane Desborough (member checking interviews): *Validation* (verification, whether as a part of the activity or separate, of the overall replication/reproducibility of results/experiments and other research outputs). Jane Desborough, Fiona Lewis, Anne Parkinson (white boarding sessions), Fiona Lewis (storyline), Jane Desborough, Fiona Lewis, Anne Parkinson (initial confirmation of framework—coffee meeting): *Formal analysis* (application of statistical, mathematical, computational or other formal techniques to analyse or synthesize study data). Fiona Lewis, Michelle Banfield (first interview), Anne Parkinson, Jane Desborough: *Investigation* (conducting a research and investigation process, specifically performing the experiments, or data/evidence collection). N/A: *Resources* (provision of study materials, reagents, materials, patients, laboratory samples, animals, instrumentation, computing resources or other analysis tools. Fiona Lewis (NVivo): *Data curation* (management activities to annotate [produce metadata], scrub data and maintain research data [including software code, where it is necessary for interpreting the data itself] for initial use and later reuse). Fiona Lewis, Jane Desborough, Anne Parkinson, Harry Ebbeck (Fiona's Medical School project): *Writing ‐ Original Draft* (preparation, creation and/or presentation of the published work, specifically writing the initial draft [including substantive translation]). Jane Desborough, Anne Parkinson, Fiona Lewis, Christine Phillips, Michelle Banfield, Harry Ebbeck: *Writing ‐ Review & Editing* (preparation, creation and/or presentation of the published work by those from the original research group, specifically critical review, commentary or revision—including pre‐ or postpublication stages). Fiona Lewis, Jane Desborough, Anne Parkinson (framework figure): *Visualisation* (preparation, creation and/or presentation of the published work, specifically visualisation/data presentation). Jane Desborough, Anne Parkinson, Michelle Banfield, Christine Phillips: *Supervision* (oversight and leadership responsibility for the research activity planning and execution, including mentorship external to the core team). Jane Desborough, Anne Parkinson, Fiona Lewis: *Project administration* (management and co‐ordination responsibility for the research activity planning and execution). OHIOH—Jane Desborough, Anne Parkinson, Christine Phillips: *Funding acquisition* (acquisition of the financial support for the project leading to this publication).

## Supporting information

Supporting information.

Supporting information.

## Data Availability

The data that support the findings of this study are available from the author on reasonable request.
