# Supplementary material for: A framework for involving coproduction partners in research about young people with type 1 diabetes
Source: Health Expect. 2021 Dec 10;25(1):430–42. doi: 10.1111/hex.13403 (PMC8849360; doi:10.1111/hex.13403)
Supplement: Supplementary file 1 — Supporting information. [file HEX-25-430-s001.docx]

Supplementary Table 1: Interview protocol for co-production partners with iterative modifications

| **Reflecting on the impact of involvement on the research**  What is your role in the research team?  What aspects of research have you been involved in? How were you involved?  What sort of things happen at the research meetings?  Have your ideas/comments been incorporated into the research? How?  *(Added to version 2) Do you think your involvement has value to it? How?*   - *Impact can be positive or negative change to the research you think your involvement has brought. Do you feel you’ve had an overall impact? How?*   *(Added to version 4) Thinking about impact and by that I mean any positive or negative changes your involvement has brought to the research, what impact do you think your involvement has had on the research?*  *Prompt if subject is wavering or uncertain of how to respond - for example, have any of your comments changed the way the research has been conducted, such as recruitment strategies? Have you contributed to specific elements of the research, such as reading papers, collecting data, analysing data, technology design?*  *(Added to version 5) Have you had feedback on how you have impacted the research? If no, would you like feedback and what kind of feedback would you like?*  **Reflecting on training for research involvement**  Have you attended any training workshops?  Did you find the workshop helpful? What did you learn?  How have you used what you learnt from workshop in the OHIOH project?  **Reflecting on the impact of involvement on you**  How has being involved in OHIOH impacted you?   - What have you learnt about research? - What have you learnt about diabetes? - How has being involved changed your thoughts/feelings about diabetes? - Has working with others that have diabetes and different management plans changed your management of your diabetes? - *How has it had an impact on you as a person?(added to version 4)*   How did you find it working in a research team?   - What sort of voice do you have in the team? - *How would you describe your role in the team?(added to version 2)* - *Are there any constraints on your role? (added to version 5)*   Do you ever feel reluctant to share ideas? Do you feel it is a safe space to share your thoughts?  *(Added to version 5) If an idea is put forward that you don’t agree with or you think there is a better solution do you feel free to challenge that idea?*   - *Does this change depending on who put the idea forward?*   Is there anything that doesn’t work, or you feel could work better?  Are there any opportunities you feel haven’t been made available yet?  **Reflecting on the six impactful roles of participants**  Do these six roles resonate with you?  Do you feel that you can relate to any in particular? How?  *Is there anything missing from these six roles. (added to version 2)*  *(Added to version 2)This process is for us to measure impact – the effect of your involvement on research and effect research has on you. Do you think this is useful? How?*  **Final Reflection**  Would you change anything you told me at the beginning after our discussion on the six roles?  In your opinion do you think there is anything else that could aid and/or optimise your contribution |
| --- |
