# Supplementary material for: A framework for involving coproduction partners in research about young people with type 1 diabetes
Source: Health Expect. 2021 Dec 10;25(1):430–42. doi: 10.1111/hex.13403 (PMC8849360; doi:10.1111/hex.13403)
Supplement: Supplementary file 2 — Supporting information. [file HEX-25-430-s002.docx]

**Researcher Interview Protocol:**

Can you tell me what your role is in the OHIOH type 1 diabetes research team?

**Reflecting on the impact of Patient and Public Involvement in research**

How do the people with type 1 diabetes (T1D) impact your research?

How do their carers impact your research?

How do the diabetes educators impact your research?

Does their involvement change the research direction/ method? How?

Has their involvement changed the way you approach meetings/ discussion about research? How?

*Can you elaborate on how their contributions at meetings have been acted on?*

Can you think of any examples of previous research about diabetes that has not involved any people with diabetes? How was the OHIOH project different to that?

**Reflecting on the impact of Patient and Public Involvement on you**

Has the involvement of people living with diabetes changed your perception of research?

Has the involvement of people living with diabetes changed your approach to research?

Has the involvement of people living with diabetes impacted you personally?

**Reflecting on the six impactful roles of PPI contributors**

Do you recognise these different roles within the people living with diabetes involved in the research team?

How do they resonate?

Is there any role the people living with diabetes play that isn’t included here? How?

**Final Reflection**

Would you change anything you told me at the beginning after our discussion on the six impactful roles?
